# Clinical Outcomes of Negative Balloon-Assisted Enteroscopy for Obscure Gastrointestinal Bleeding: A Systematic Review and Meta-Analysis

**DOI:** 10.3389/fmed.2022.772954

**Published:** 2022-03-04

**Authors:** Xiao Dong Shao, Hao Tian Shao, Le Wang, Yong Guo Zhang, Ye Tian

**Affiliations:** ^1^Department of Gastroenterology, General Hospital of Northern Theater Command, Shenyang, China; ^2^School of Basic Medical Sciences, Guangxi Medical University, Nanning, China

**Keywords:** obscure gastrointestinal bleeding, small intestine, enteroscopy, rebleeding, follow-up

## Abstract

**Background:**

For patients with obscure gastrointestinal bleeding (OGIB), finding the bleeding site is challenging. Balloon-assisted enteroscopy (BAE) has become the preferred diagnostic modality for OGIB. The long-term outcome of patients with negative BAE remains undefined. The present study aimed to evaluate the long-term outcomes of patients with negative BAE results for OGIB and to clarify the effect of further investigations at the time of rebleeding with a systematic review and meta-analysis of the available cohort studies.

**Methods:**

Studies were searched through the PubMed, EMBASE, and Cochrane library databases. The following indexes were analyzed: rebleeding rate after negative BAE, rebleeding rate after different follow-up periods, the proportion of patients who underwent further evaluation after rebleeding, the percentage of patients with identified rebleeding sources, and the percentage of patients with rebleeding sources in the small intestine. Heterogeneity was assessed using the I^2^ test.

**Results:**

Twelve studies that involved a total of 407 patients were included in the analysis. The pooled rebleeding rate after negative BAE for OGIB was 29.1% (95% CI: 17.2–42.6%). Heterogeneity was significant among the studies (I^2^ = 88%; *p* < 0.0001). The Chi-squared test did not show a difference in rebleeding rates between the short and long follow-up period groups (*p* = 0.142). The pooled proportion of patients who underwent further evaluation after rebleeding was 86.1%. Among the patients who underwent further evaluation, rebleeding sources were identified in 73.6% of patients, and 68.8% of the identified rebleeding lesions were in the small intestine.

**Conclusion:**

A negative result of BAE in patients with OGIB indicates a subsequently low risk of rebleeding. Further evaluation should be considered after rebleeding.

## Introduction

The small intestine has always been difficult to evaluate thoroughly because of its long length and variable looped configuration. Before balloon-assisted enteroscopy (BAE) was introduced in clinical practice in 2001, endoscopic examination of the small intestine was unsatisfactory, and treatment of small intestinal disease often required surgical laparotomy with intraoperative enteroscopy. The development of BAE had made the entire small intestine accessible to endoscopic observation. BAE can achieve a complete small intestinal examination by using one (single balloon enteroscopy, SBE) or two balloons (double balloon enteroscopy, DBE) to fix the intestinal wall and to facilitate endoscopic intubation in the small intestine (1). The most common indication of BAE is obscure gastrointestinal bleeding (OGIB), which is defined as bleeding of an unknown origin despite traditional endoscopy (2). OGIB accounts for ~1.2–5% of all GI bleeding events (3, 4) and can be further classified as overt or occult OGIB. Obscure-overt GI bleeding in patients presents as clinically visible bleeding, such as hematemesis, melena, or hematochezia (5, 6). In contrast, the occult type presents as iron deficiency anemia or a positive occult blood test in the stool (7, 8). Most OGIB events are attributable to small intestinal diseases. BAE has become the preferred method for examination of the small intestine in OGIB. BAE can examine much more of the small intestine compared with push enteroscopy and can achieve a much higher diagnostic yield (9, 10). The diagnostic yields of BAE for patients with OGIB have been reported as 43–81% (11–20), but the long-term outcome of patients with OGIB after BAE has been indeterminate. A prospective study of patients with OGIB who underwent BAE showed reduced bleeding and blood transfusion (21). However, some follow-up studies found a rebleeding rate ranging from 40 to 46% in patients with OGIB who are treated with BAE (22–24).

For some patients with OGIB, the bleeding source may not be found with BAE. It is believed that the prognosis of patients with identified bleeding sites responsible for OGIB was better than that of patients with a negative result. Studies about capsule endoscopy (CE) in OGIB reported rebleeding rates ranging from 5 to 53% after negative CE (25–30). The long-term outcomes of positive BAE results for small intestinal bleeding have been investigated in some studies and the rebleeding rates are between 10 and 50% (31–37). There are a few reports about the outcomes of patients after negative BAE. A few small studies have evaluated the rebleeding rate after negative BAE, and the reported rebleeding rates range from 33.3 to 55.9% (38–41). The long-term outcome of patients with negative BAE is not clear until now, and the role of a further evaluation at the time of rebleeding needs to be investigated. The optimal management strategy for patients with negative findings of initial BAE remains elusive. The present study attempts to investigate the long-term outcomes of OGIB patients with negative BAE and to evaluate the role of further examinations at the time of rebleeding with a systematic review and meta-analysis of available cohort studies.

## Methods

### Literature Search

Relevant studies were identified by searching in the PubMed, EMBASE, and Cochrane Library databases from January 2001 to December 2020. Search items were listed as follows: “obscure gastrointestinal bleeding” or “OGIB” or “small intestinal bleeding” or “small bowel bleeding” and “double balloon enteroscopy” or “single balloon enteroscopy” or “balloon-assisted enteroscopy” and “negative” or “normal”, and “follow-up”. The search was limited to studies in humans published in English. References of eligible articles and review articles were manually searched.

### Selection of Articles

The selection criteria were studies in (1) patients who underwent DBE or SBE due to OGIB; (2) initial BAE results were negative; (3) the patients were followed up; and (4) the rebleeding rates after negative BAE were presented. The exclusion criteria were abstracts, reviews and meta-analyses, editorials, case reports, and studies that did not report the rebleeding rate. Each eligible article was reviewed in full text. Two reviewers (SHT and ZYG) independently performed literature search and then cross-checked the search results. Two authors (SHT and ZYG) fulfilled study selection and data extraction and a third reviewer (SXD) was involved if there was any conflict.

### Data Extraction

The following data were extracted from all eligible studies: author, country, publication year, publication type, study design, type of BAE used, the number of patients with negative BAE, the number of patients with rebleeding, the number of patients who underwent further evaluation, the number of patients whose rebleeding source was identified in the further evaluation, the number of patients whose identified lesion was in the small intestine, and the length of follow-up.

### Definitions

Negative BAE: No obvious cause of blood loss was identified during BAE.

Rebleeding: Evidence of GI bleeding was found at least 30 days after an initial BAE.

Long-term follow-up: A follow-up continued for ≥2 years after BAE.

Short-term follow-up: A follow-up continued for <2 years after BAE.

### Risk of Bias and Publication Bias Analysis

The risk of bias was assessed by the Newcastle-Ottawa Scale (NOS) criteria for cohort studies. There are three major parts assessed: (1) selection (score 0–4); (2) comparability (score 0–2); and (3) outcome (score 0–3). The maximum score is 9. A score of 0–3, 4–6, and 7–9 represents a low, moderate, and high quality, respectively. The publication bias was assessed by the Egger test.

### Statistical Analysis

A random-effects model with StatsDirect statistical software Version 2.7.8 (StatsDirect Ltd., Sale, Cheshire, UK) was performed in all analyses to generate a more conservative estimate. We pooled proportions with 95% CIs, which are presented as forest plots. The heterogeneity between studies was estimated by the Cochran Q test and the I^2^ statistics. *p* < 0.1 and I^2^ > 50% were considered to be significantly heterogeneous. Categorical variables are presented as absolute numbers and percentages. Statistically significant differences were evaluated using the Chi-squared test for categorical variables. Results were considered as significant at *p* < 0.05.

## Results

### Literature Search Results

**Figure 1:**
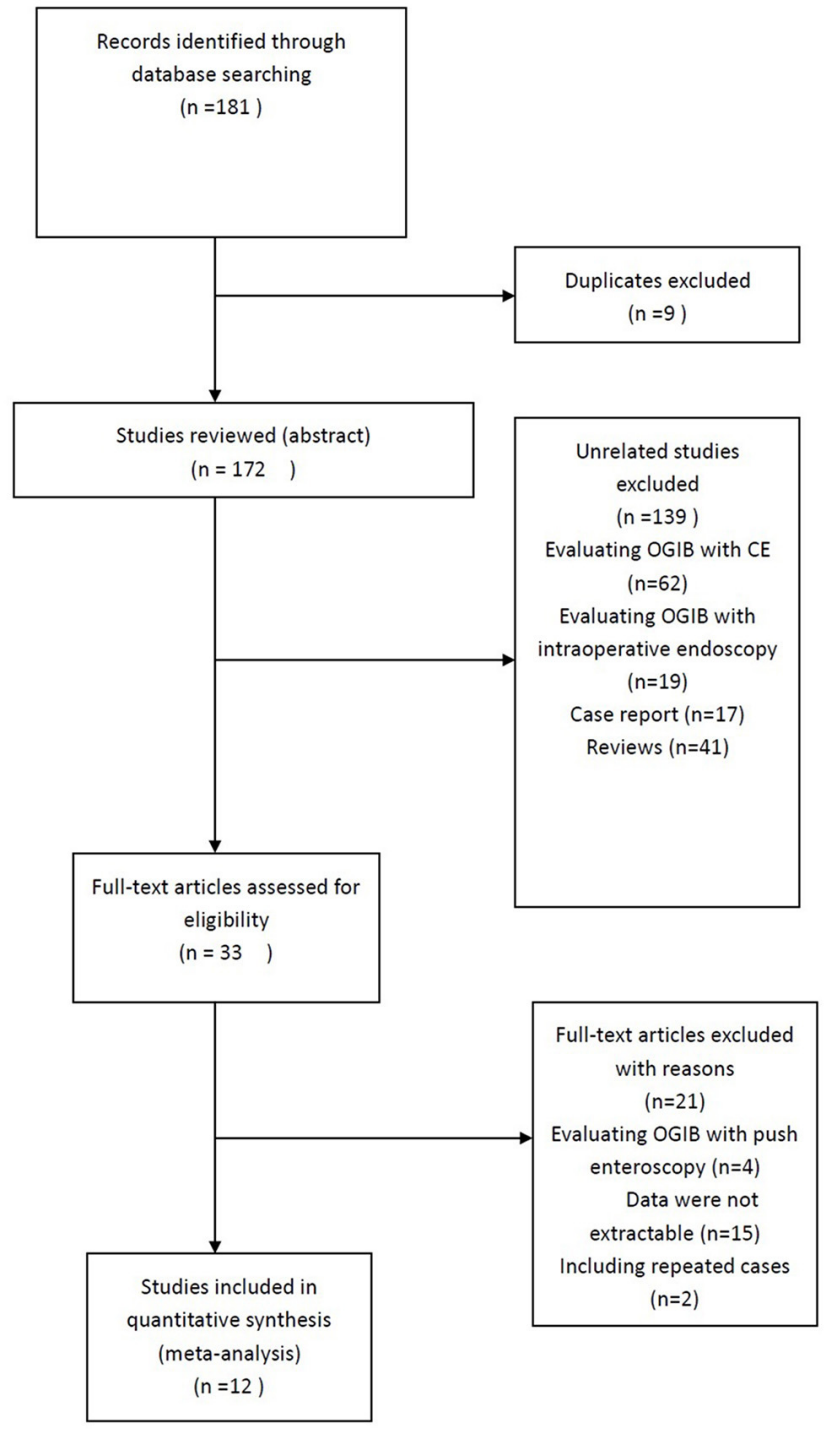
Study selection flow chart. Of a total of 181 studies, only 12 studies met selection criteria.

Twelve studies that involved a total of 407 patients were included in the analysis. All studies were retrospective and published between 2007 and 2020. The results of the literature search are summarized in Figure 1. The characteristics of the 12 eligible studies are summarized in Table 1.

**Table 1:** Study characteristics.

**Author**	**Year**	**Country**	**Publication type**	**Study type**	**BAE used in study**	**No. of cases**
Fujimori (42)	2007	Japan	Fulltext	Retrospective	DBE	18
Hsu (43)	2007	Taiwan	Fulltext	Retrospective	DBE	5
Madisch (44)	2008	Germany	Fulltext	Retrospective	DBE	22
Arakawa (18)	2009	Japan	Fulltext	Retrospective	DBE	52
Gerson (24)	2009	USA	Fulltext	Retrospective	DBE	43
Fujita (45)	2010	Japan	Fulltext	Retrospective	DBE	47
Shishido (46)	2012	Japan	Fulltext	Retrospective	DBE	26
Kushnir (41)	2013	USA	Fulltext	Retrospective	SBE	34
Shinozaki (39)	2015	Japan	Fulltext	Retrospective	DBE	42
Hashimoto (40)	2018	Japan	Fulltext	Retrospective	DBE	63
Zhao (47)	2020	China	Fulltext	Retrospective	DBE	20
Gomes (48)	2020	Spain	Fulltext	Retrospective	SBE and DBE	35

### Characteristics of Study

In the 12 studies, a total of 407 patients underwent BAE procedures for OGIB with negative results. All studies were conducted between 2007 and 2020. The included 12 studies were retrospective, six of which were performed in Japan, followed by the United States (2/12), China (1/12), Germany (1/12), Spain (1/12), and Taiwan (1/12). The number of patients in each eligible study was more than 5 and the largest one included 63 patients. In one study, only SBE was used (41) and both SBE and DBE were adopted in another study (48). In the other 10 studies, only DBE was performed. Six studies had a long duration of follow-up (2 years or more). The results of the various outcomes of the individual studies are shown in Table 2.

**Table 2:** Outcomes of the individual studies.

**Author**	**Negative BAE**	**Rebleeding after negative BAE**	**Follow up time**	**Patients undergoing further evaluation**	**Identified rebleeding source in further evaluation**	**Identified lesions located in small intestine**
Fujimori et al. (42)	18	3 (16.7%)	Short	NA	NA	NA
Hsu et al. (43)	5	4 (80%)	Short	NA	NA	NA
Madisch et al. (44)	22	4 (18.2%)	Short	NA	NA	NA
Arakawa et al. (18)	52	0 (0%)	Short	NA	NA	NA
Gerson et al. (24)	43	18 (41.9%)	Long	NA	NA	NA
Fujita et al. (45)	47	12 (25.5%)	Long	NA	NA	NA
Shishido et al. (46)	26	1 (3.8%)	Long	1	1	1
Kushnir et al. (41)	34	19 (55.9%)	Short	13	5	4
Shinozaki et al. (39)	42	16 (38.1%)	Long	14	10	7
Hashimoto et al. (40)	63	21 (33.3%)	Long	21	19	15
Zhao et al. (47)	20	8 (40%)	Short	6	4	2
Gomes et al. (48)	35	14 (40%)	Long	12	11	6

### Risk of Bias and Publication Bias Analysis

The NOS score ranged from 6 to 8 points. Six studies were considered to be of moderate quality, and 6 were of high quality (41–43, 45–47) (Supplementary Table S1). Publication bias (Egger test): 4.967 (95% CI = 3.259–6.674); *p* < 0.0001.

### Rebleeding Rate After Negative BAE

**Figure 2:**
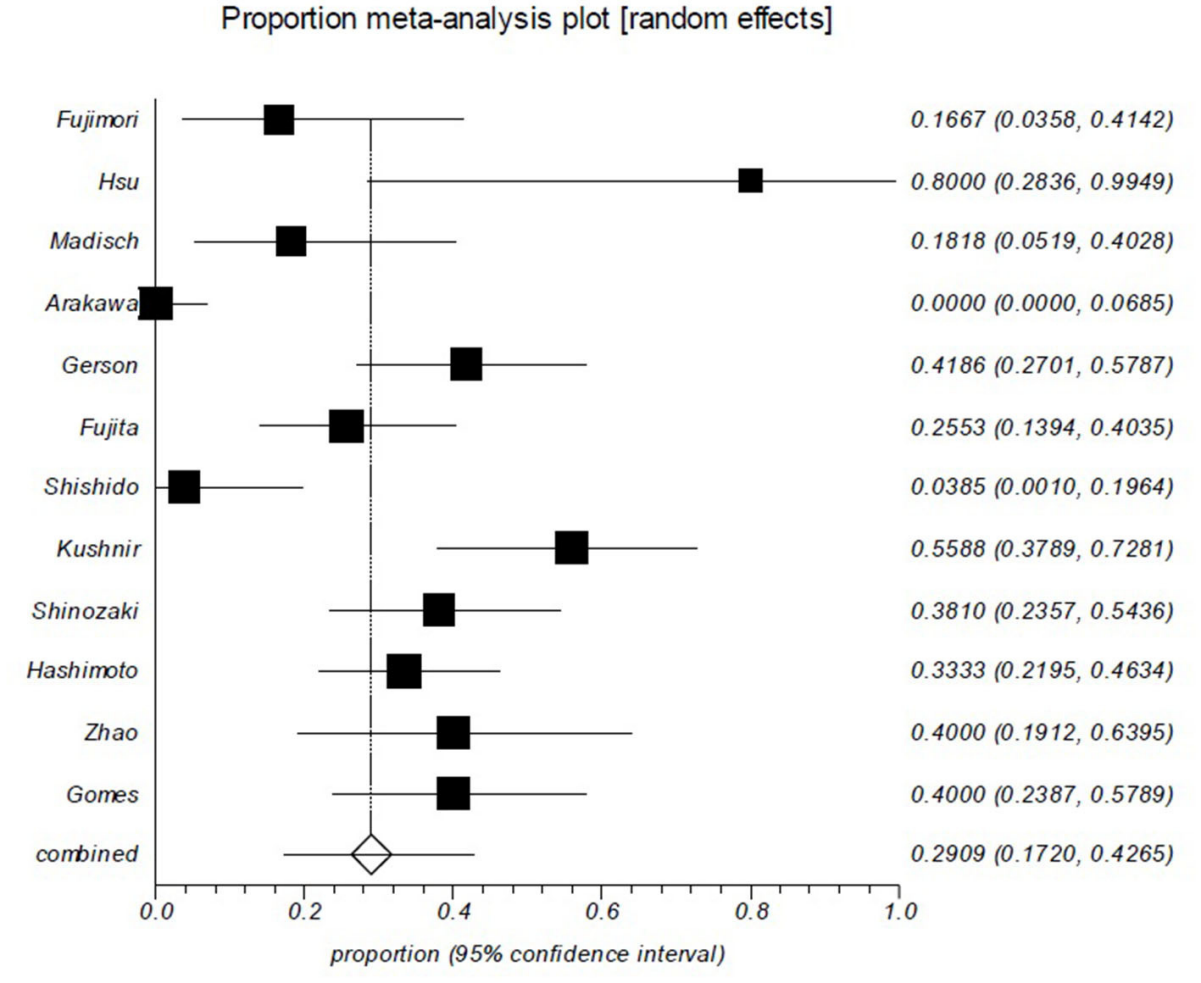
Rebleeding rate after negative balloon-assisted enteroscopy (BAE) is ~ 29%. Rebleeding in patients after negative BAE for obscure gastrointestinal bleeding (OGIB). Rebleeding episodes during the follow-up period were reported in 29.1% (95% CI: 17.2–42.6%) of the 407 patients in the 12 studies. There was significant heterogeneity among the studies (*p* < 0.0001).

The rebleeding rates of all included studies ranged from 0 to 80%. The overall, pooled rebleeding rate after negative BAE for OGIB was 29.1% (95% CI: 17.2–42.6%; Figure 2). There was significant heterogeneity among the studies (I^2^ = 88%; *p* < 0.0001). One study where only SBE was used in all patients had a higher rebleeding rate (55.9%) (41).

### Rebleeding Rate After a Different Follow-Up Period

**Figure 3:**
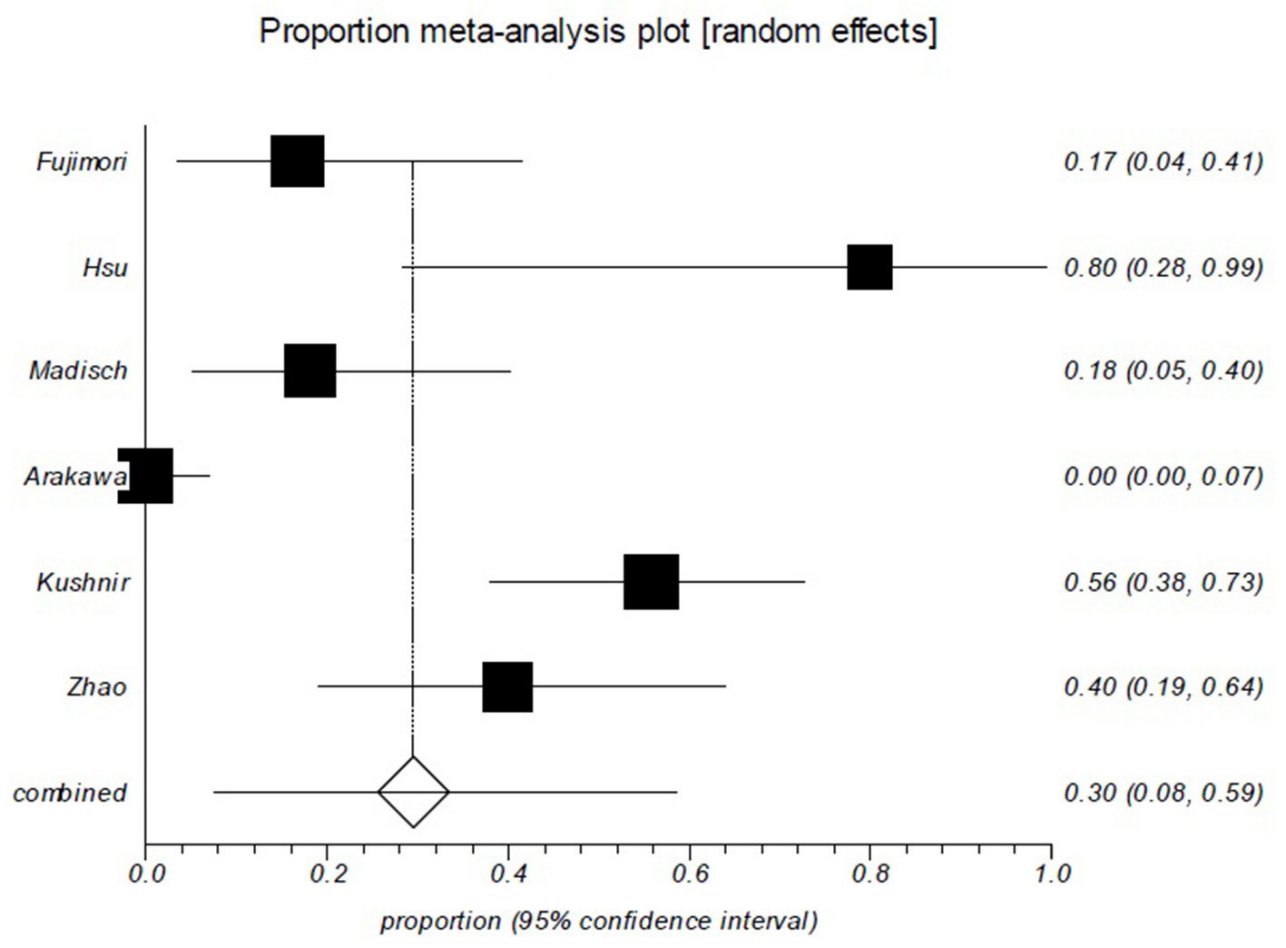
Rebleeding rate of patients in the short follow-up group is ~30%. Forest plot shows that 29.6% (95% CI: 7.5–58.6%) of the patients who had been followed up less than 2 years experienced rebleeding. There was evidence of heterogeneity among the studies (*p* < 0.0001).

**Figure 4:**
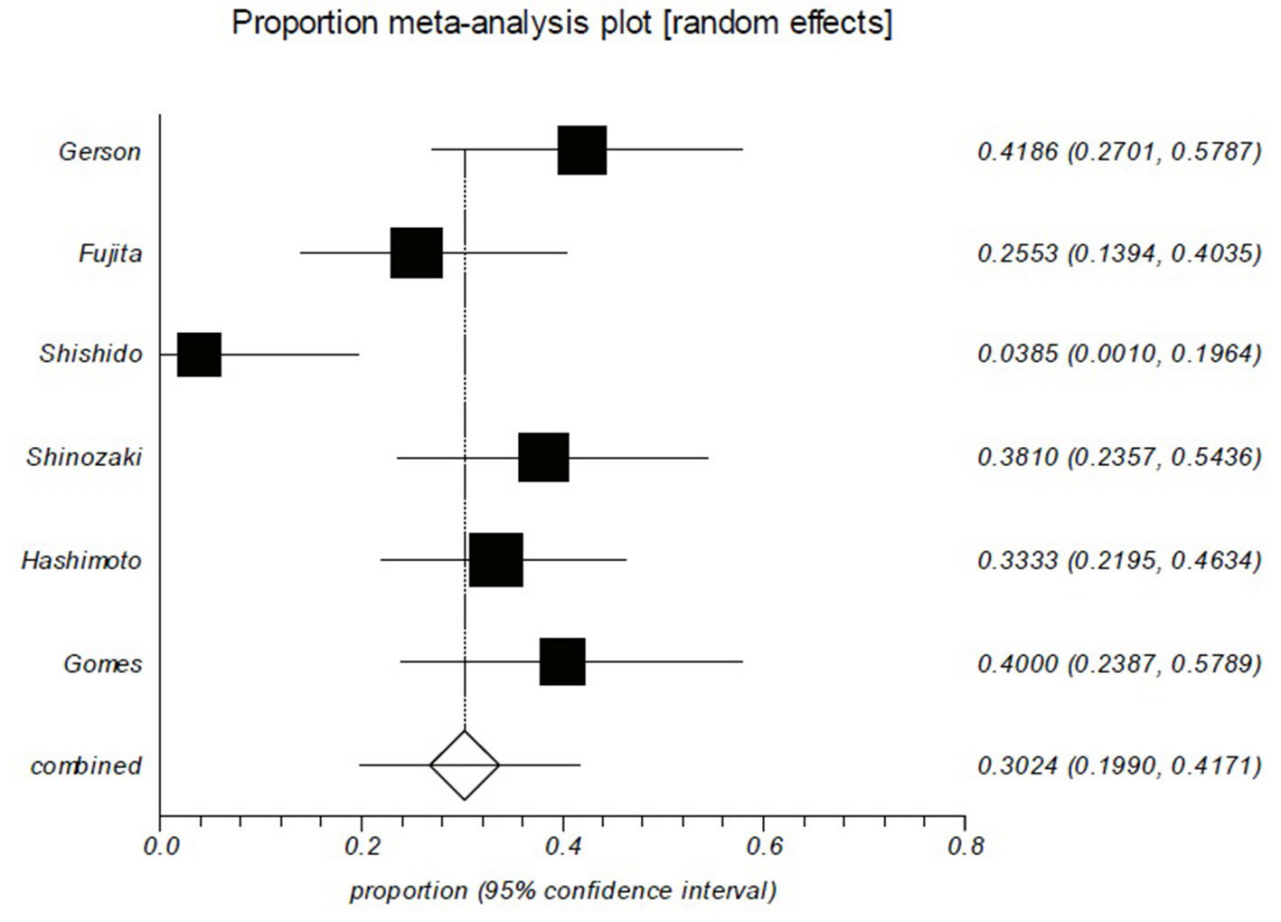
Rebleeding rate of patients in the long follow-up group is ~30%. Forest plot shows that 30.2% (95% CI: 19.9–41.7%) of the patients who had been followed up more than 2 years experienced rebleeding. There was evidence of heterogeneity among the studies (*p* = *0.0018*).

As shown in Table 2, the rebleeding rates in the short follow-up period group were ranged from 0 to 80%. The pooled rebleeding rate was 29.6% (95% CI: 7.5–58.6%) in this group (Figure 3). Significant heterogeneity was found among the studies (I^2^ = 92.4%; *p* < 0.0001). The rebleeding rates in the long follow-up period group ranged from 3.8 to 41.9%, and the pooled rebleeding rate was 30.2% (95% CI: 19.9–41.7%; Figure 4). Heterogeneity was significant among the studies (I^2^ = 73.9%; *p* = 0.0018). The Chi-squared test did not show a difference in rebleeding rates between the short and long follow-up period groups (*p* = 0.142).

### Further Evaluation After Rebleeding

Six studies provided the data of patients who underwent further evaluation after rebleeding (Table 2). The pooled proportion of patients who underwent further evaluation after rebleeding was 86.1% (95% CI: 74.4–97.9%). Among the patients who underwent further evaluation, the rebleeding sources were identified in 73.6% (95% CI: 54.9–88.7%) of patients, and 68.8% (95% CI: 56.1–80.1%) of the identified rebleeding lesions were in the small intestine.

## Discussion

The introduction of BAE has improved the diagnostic yield for patients with OGIB. However, some small intestinal lesions can be missed during BAE and false-negative BAE results for OGIB are not rare. The delayed BAE may be performed when the original bleeding site had healed. Some lesions were self-limiting and experienced a rapid resolution. When the etiologic factors were addressed, a prompt stop of bleeding could be expected in some cases. The timing of BAE could contribute to improving the diagnostic yield of patients with OGIB. Some studies have shown that emergency DBE is associated with a lower rebleeding rate compared with non-emergency DBE (49, 50). A study demonstrated that emergency DBE is helpful for the diagnosis and management of patients with small intestinal bleeding (51). Therefore, some experts suggested that BAE should be performed within the first few days after OGIB. Some small intestinal lesions found with additional diagnostic modalities after initial negative BAE were not within the reach of the first BAE. This was due to an insufficient insertion depth of the first procedure. In comparison with CE and DBE, CT has a lower diagnostic yield for OGIB (52). However, a study showed that CT enterography found the lesion in 50% of OGIB cases with negative CE results (53). A meta-analysis indicated that urgent CT angiography could localize the bleeding lesion of OGIB patients with high sensitivity and specificity (54). Based on these reports, the concomitant use of CT examination could also improve the detection of bleeding sources missed during BAE.

Rebleeding episodes can occur many years after the initial negative BAE results. In the present study, we found that the pooled rebleeding rate after an initial negative BAE was 29.1%. This is similar to the rebleeding rate after positive DBE (31–33). The rebleeding rates after negative BAE varied from 0 to 80% among included studies. The severity of OGIB in these reports was different. A study had found that the severity of OGIB is a key factor of the long-term outcomes after a DBE with negative results (39). The degree of OGIB may explain the differences in the reported rebleeding rates after a negative BAE. The follow-up period in these reports is also variable and may have an effect on the clinical outcomes. However, there was no difference in rebleeding rate between the long and short follow-up period groups in our study (30.2 vs. 29.6%, *p* = 0.142). Some reports estimated that, in 5–53% of patients with negative CE, rebleeding is expected (25, 26, 29). Other studies suggested that a negative CE could reliably predict a low rebleeding rate in the future (28, 55). More than 70% of OGIB patients with negative BAE had no rebleeding during the follow-up in the present study. This means that a close follow-up for patients with negative BAE may be appropriate. Our results support a watch-and-wait policy that does not recommend a second-look BAE unless there is strong evidence of rebleeding.

In the present study, 86.1% of patients with rebleeding underwent further evaluation, such as BAE repetition, CE, and CT, and the identification of the bleeding source was achieved in 73.6% of these patients. Currently, there are no guidelines to clarify the preferred diagnostic modality after a rebleeding event in a patient with a previously negative BAE. When an episode of rebleeding occurs, prompt endoscopy examination may find the bleeding site and allow therapeutic interventions in some cases. For patients who have rebleeding after OGIB episode, BAE is safe and effective to detect the origin of bleeding. Repeated BAE should be considered when a patient has rebleeding with negative BAE results during previous bleeding. In fact, in our study, 68.8% of the identified rebleeding lesions were in the small intestine. A thorough endoscopic examination could improve clinical outcomes in these patients. Apparently, in the presence of a rebleeding episode, the use of alternative non-invasive methods, such as CE and CT, may also contribute to lesion detection. Although CT usually has a lower diagnostic yield for OGIB compared to CE or BAE, it has already been shown that a repeat CT at the time of rebleeding may help to identify the source of rebleeding.

There are some limitations in the present study. The heterogeneity of the studies was significant. Much of the literature mixes patients with overt and occult obscure GI bleeding. These patients should be analyzed separately because they may have different prognoses. Many of the included studies did not intentionally evaluate outcomes after negative BAE. A study showed that enteroscopy with DBE had a higher complete enteroscopy rate and a higher diagnostic yield compared with SBE (56). Another report demonstrated that the complete enteroscopy rate is higher for DBE than for SBE (11). Most of the included studies adopted DBE to investigate OGIB, but in two studies, SBE was used which may influence the analyzing results.

In conclusion, the results of this meta-analysis may help decide the management of patients with OGIB and negative BAE. Our analysis shows that a negative BAE in patients with OGIB implies a subsequently low risk of rebleeding. Such patients can be closely followed up without further evaluation unless there is a rebleeding episode. Further investigation should be considered after rebleeding even if the initial BAE results are negative, as the diagnostic yield of further evaluation is more than 70% in these patients.

## Data Availability Statement

The raw data supporting the conclusions of this article will be made available by the authors, without undue reservation.

## Author Contributions

XDS: conception and design and administrative support. YT, LW, and XDS: provision of study materials or patients. HTS, YGZ, LW, and XDS: collection and assembly of data, data analysis, and interpretation. HTS, YGZ, LW, YT, and XDS: manuscript writing and final approval of manuscript. All authors contributed to the article and approved the submitted version.

## Conflict of Interest

The authors declare that the research was conducted in the absence of any commercial or financial relationships that could be construed as a potential conflict of interest.

## Publisher's Note

All claims expressed in this article are solely those of the authors and do not necessarily represent those of their affiliated organizations, or those of the publisher, the editors and the reviewers. Any product that may be evaluated in this article, or claim that may be made by its manufacturer, is not guaranteed or endorsed by the publisher.
