# Supplementary material for: Clinical Outcomes of Negative Balloon-Assisted Enteroscopy for Obscure Gastrointestinal Bleeding: A Systematic Review and Meta-Analysis
Source: Front Med (Lausanne). 2022 Mar 4;9:772954. doi: 10.3389/fmed.2022.772954 (PMC8931682; doi:10.3389/fmed.2022.772954)
Supplement: Supplementary file 1 [file Table_1.docx]

| **Supplementary table 1. Quality of included studies** | | | | | | | | | | |
| --- | --- | --- | --- | --- | --- | --- | --- | --- | --- | --- |
| **Studies** | **Quality Score** | **Selection (☆☆☆☆)** | | | | **Comparability (☆☆)** | | **Outcome (☆☆☆)** | | |
| Fujimori(42) | 7 | ☆ | ☆ | ☆ | ☆ | / | / | ☆ | ☆ | ☆ |
| Hsu(43) | 8 | ☆ | ☆ | ☆ | ☆ | / | ☆ | ☆ | ☆ | ☆ |
| Madisch(44) | 6 | ☆ | ☆ | ☆ | ☆ | / | ☆ | ☆ | / | / |
| Arakawa(18) | 6 | ☆ | ☆ | ☆ | ☆ | / | / | ☆ | / | ☆ |
| Gerson(24) | 6 | ☆ | ☆ | ☆ | ☆ | / | / | / | ☆ | ☆ |
| Fujita(45) | 7 | ☆ | ☆ | ☆ | ☆ | / | ☆ | ☆ | / | ☆ |
| Shishido(46) | 8 | ☆ | ☆ | ☆ | ☆ | ☆ | / | ☆ | ☆ | ☆ |
| Kushnir(41) | 8 | ☆ | ☆ | ☆ | ☆ | / | ☆ | ☆ | ☆ | ☆ |
| Shinozaki(39) | 6 | ☆ | / | ☆ | ☆ | / | / | ☆ | ☆ | ☆ |
| Hashimoto(40) | 6 | ☆ | ☆ | ☆ | ☆ | / | / | ☆ | ☆ | / |
| Zhao(47) | 8 | ☆ | ☆ | ☆ | ☆ | / | ☆ | ☆ | ☆ | ☆ |
| Gomes(48) | 6 | ☆ | / | ☆ | ☆ | / | / | ☆ | ☆ | ☆ |
| **Notes:** / indicates no star | | | | | | | | | | |
